# Phylogenetic signal in the vocalizations of vocal learning and vocal non-learning birds

**DOI:** 10.1098/rstb.2020.0241

**Published:** 2021-10-25

**Authors:** Jozsef Arato, W. Tecumseh Fitch

**Affiliations:** ^1^ Vienna Cognitive Science Hub, University of Vienna, Vienna, Austria; ^2^ Department of Cognitive Biology, University of Vienna, Vienna, Austria

**Keywords:** vocal learning, birdsong, bioacoustics, phylogenetic signal, evolution of communication

## Abstract

Some animal vocalizations develop reliably in the absence of relevant experience, but an intriguing subset of animal vocalizations is learned: they require acoustic models during ontogeny in order to develop, and the learner's vocal output reflects those models. To what extent do such learned vocalizations reflect phylogeny? We compared the degree to which phylogenetic signal is present in vocal signals from a wide taxonomic range of birds, including both vocal learners (songbirds) and vocal non-learners. We used publically available molecular phylogenies and developed methods to analyse spectral and temporal features in a carefully curated collection of high-quality recordings of bird songs and bird calls, to yield acoustic distance measures. Our methods were initially developed using pairs of closely related North American and European bird species, and then applied to a non-overlapping random stratified sample of European birds. We found strong similarity in acoustic and genetic distances, which manifested itself as a significant phylogenetic signal, in both samples. In songbirds, both learned song and (mostly) unlearned calls allowed reconstruction of phylogenetic trees nearly isomorphic to the phylogenetic trees derived from genetic analysis. We conclude that phylogeny and inheritance constrain vocal structure to a surprising degree, even in learned birdsong.

This article is part of the theme issue ‘Vocal learning in animals and humans’.

## 1. Introduction

The fact that closely related species resemble one another in form is a fundamental insight in evolutionary biology [1–3], and the realization that the same is often true of behavioural patterns was foundational in the development of ethology [4–6]. Vocalizations produced by a given species reflect both vocal anatomy and behaviour, each of which may have strong heritable components. This suggests that homologous vocalizations of closely related species may resemble each other more than pairs drawn at random—in other words that vocalizations may possess ‘phylogenetic signal’.

Indeed, the pioneering work of Konrad Lorenz found strong phylogenetic constraints on the display vocalizations of ducks [4], and significant phylogenetic signal in vocalizations has since been demonstrated in a variety of clades. However, much previous work has focused on calls with a strong innate basis, raising the question of whether phylogenetic signal persists in vocalizations with a strong learned component. On the one hand, since vocally learned signals are flexible and can change rapidly over generations [7], we might predict little or no phylogenetic signal in learned vocalizations. However, to the extent that learned vocalizations reflect inherited constraints imposed by species-typical motor circuitry or vocal anatomy, and/or biases in the learning mechanism itself, they might still possess phylogenetic signal, even if at reduced levels.

**Figure 1.**
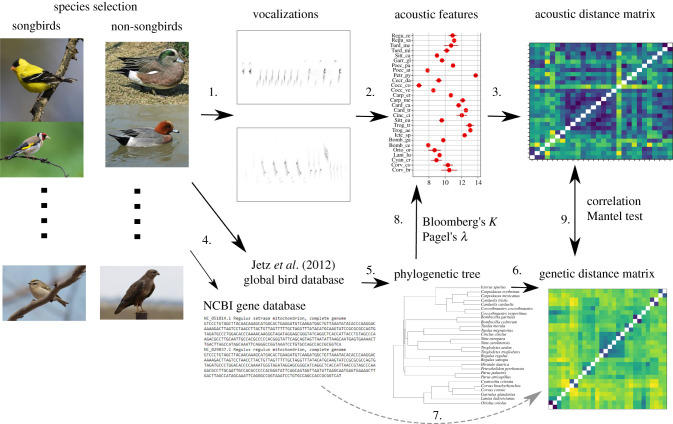
Method overview. Step 1. We start by making a selection of passerine and non-passerine species (pictures: example species) for which high-quality vocalization recordings are available. 2. Calculate acoustic features for each vocalization. 3. Calculate the acoustic distance matrix. 4. Obtain bootstrapped phylogenetic tree information and download mitochondrial genome. 5. Calculate consensus tree for predicting acoustic feature values with phylogenetic methods (8). Obtain genetic distance matrix from bootstrapped trees (6) and based on mitochondrial *ND2* gene sequences (7). 9. Compare acoustic and genetic distance matrices with various statistical tests. (Online version in colour.)

Most previous phylogenetic work on vocalizations has concentrated either on a small set of species (e.g. [8–11]) or, in the few broad-scale studies available, on innately determined calls [12–17]. Nonetheless, these studies indicate that phylogenetic signal is present in vocalizations in some clades of frogs, mammals and birds. In some cases, these studies also provide indications of what aspects of vocalizations have a strong phylogenetic signal. For example, evolution was slow for aspects of calls tied to vocal anatomy in chorus frogs (*Hyla*), but more rapid for behavioural aspects like call rate [18]. In birds, similar results were found for the unlearned calls of herons [8].

A smaller set of studies has revealed phylogenetic signal in the songs of specific genera of vocal learning birds [9–11], suggesting that phylogeny may continue to play a constraining role even in learned signals. For example, Päckert *et al.* analysed song evolution in the six species of the genus *Regulus* (crests and kinglets) using character tracing on a molecular phylogeny and concluded that both song syntax and frequency parameters contained significant phylogenetic signal in this group [9]. Similarly, Price & Lanyon [10,11] found that song evolution in the oropendolas is surprisingly evolutionarily conservative. These results suggest that phylogenetic signal persists even in passerines that learn their songs.

In the broadest-scale study to date, Medina-Garcia and colleagues [19] explicitly considered whether vocal learning eliminates phylogenetic signal in contact calls in 51 New World parrot species of the tribe Arini. Parrots are open-ended learners with a wide variety of body sizes and occupying diverse habitats, making them an excellent group to evaluate this issue. The authors found clear evidence of preserved phylogenetic signal in this clade, even when controlling for habitat type and two morphological characteristics (body size and bill length). They concluded that acoustic parameters of these neotropical parrots are highly conserved across evolution, despite clear evidence for vocal learning.

These previous studies strongly suggest that phylogenetic signal can persist in avian vocalizations, whether or not the clade consists of vocal learning species. However, none of these previous studies made direct comparisons between vocal learners and non-learners, or between learned and unlearned vocalizations in a vocal learning clade. In the current study (figure 1), we assess phylogenetic signal in bird vocalizations, analysing calls and songs produced by a wide phylogenetic range of bird species. Our database includes both vocal learners and species with mostly ‘innate’ calls (reliably developing in the absence of a model vocalization) and considers both acoustic and genetic data. We first developed our methods using a hand-chosen database of bird species pairs, mostly congeners from Europe and North America, to gain insight into appropriate methodology and parameters. Then, we applied these tools to a random stratified sample of vocalizations from European species, contrasting vocal learners from this region (passerines of the suborder Passeri, a.k.a. oscine passerines or ‘songbirds’ hereafter) against vocal non-learners, and within songbirds contrasting calls, which are predominantly innate, with learned songs. Our goal was to provide a first quantitative comparison, across multiple avian orders, of phylogenetic signal in learned and unlearned signals. Based on the logic and the previous findings above, our predictions were that even learned vocalizations will possess significant phylogenetic signal, but that this signal may be lower in learned than unlearned vocalizations.

Many previous studies of this issue used customized acoustic parameters chosen for their specific applicability to the clade under investigation, such as fundamental frequency, or duration of an initial whistle. Because our sample of species is very broad phylogenetically, and such bespoke parameters apply only to a specific clade, in our case we used simple and broadly applicable acoustic parameters that could be reliably extracted from most vocal samples (such as spectral centroid—simply the centre of mass of all frequency bands present in a vocalization—or spectral flux—the change across temporal bins in frequency content). These parameters were based on earlier work on frog vocalizations [13] and allowed us to cast a very wide net in terms of the species samples we analysed.

## 2. Methods

### (a) Datasets

We worked with two datasets. The first ‘congener’ dataset was hand-chosen and used to develop and analyse our methods, while the second ‘test’ dataset was randomly selected. The congener dataset involved pairs of closely related North American and European species, mostly congeners. Our reasoning was that using close relatives would provide an unambiguous close match genetically, while choosing species from separate continents should minimize the degree of recent gene flow and/or ‘cultural’ flow [20] between each pair. The rationale behind this dataset was that the presence of close relatives should increase our chances of detecting phylogenetic signal for this dataset. The use of congeners also allowed a relatively straightforward selection of behaviourally homologous vocalizations from each pair. The use of homologous vocalizations is important because (for example) comparing alarm calls of one species with songs of another could artificially inflate acoustic differences [6,10,11,21]. This dataset included 28 passerine and 26 non-passerine species.

For the second ‘random’ dataset, we generated a stratified random sample based on a very large published collection of European bird vocalizations [22]. This collection included more than 2800 recordings of 800 species whose ranges included Europe, North Africa and western Asia, as well as occasional North American species; it was chosen for its high recording quality and unambiguous species identification, and because it contained recordings of multiple vocalization types for each species (including both songs and calls for most songbirds). All passerines in this collection are oscine passerines (songbirds), where evidence for vocal learning is strong in most clades. Because no other known vocal learning clades (parrots or hummingbirds) were analysed here, we assume hereafter that all analysed passerine species were vocal learners, and non-passerines were not. We thus used custom Python code to select a random sample, stratified to 47 passerines versus 40 non-passerines, and within passerines to calls (37 species) versus songs (43 species), and excluding species present in our initial training dataset. We combined our acoustic analyses with previously published and verified phylogenies [23,24] to calculate genetic distances and phylogenetic signal in this second sample. Here, we can assume an accurate phylogeny, as well as a wide distribution of phylogenetic differences, but there were many fewer closely related species. To the extent that genetic determinants of vocal acoustics evolve rapidly, this dataset should yield fewer close matches and thus lower estimates of phylogenetic signal.

Finally, to gain more power, we created ‘combined’ datasets by merging all songbird species with songs available from the congener and the random sample into a combined dataset, and all non-songbird species into a combined non-songbird dataset (table 1).

**Table 1.** Dataset information.

dataset	no. species	no. vocalizations/species mean ± s.d.)	total no. vocalizations	vocalization length, mean ± s.d. (s)
congener set	54	4.11 ± 1.38	222	2.33 ± 2.56
random songbird songs	43	4.42 ± 1.38	190	3.27 ± 3.59
random songbird calls	37	3.73 ± 1.06	138	0.49 ± 0.28
random non-songbirds	40	4.0 ± 1.32	160	2.16 ± 2.81
combined songbird songs	71	4.44 ± 1.4	315	2.84 ± 2.95
combined non-songbirds	66	3.89 ± 1.29	257	2.3 ± 3.12

### (b) Genetic distance and phylogenetic tree analysis

To calculate an estimate of the genetic distances for the congener sample, we queried the NCBI gene database (https://www.ncbi.nlm.nih.gov/gene) for the sequence of the mitochondrial NADH dehydrogenase subunit 2 (*ND2*) gene using the Entrez module of the BioPython package. *ND2* has been frequently used to estimate the genetic distance between avian species [25,26]. *ND2* sequence was available and hence downloaded for 54 bird species including 28 passerine and 26 non-passerine species (from an initial list of 30 passerine and 30 non-passerine species). Whenever possible, we downloaded the mitochondrial genome sequences from the NCBI RefSeq database; when there was no sequence for a given species in the RefSeq database, we used another *ND2* sequence from the NCBI gene database. Next, we aligned the downloaded sequences using *ClustalW* and calculated the pairwise genetic distance between the aligned sequences using the *DistanceCalculator* function from the package *Phylo* module of *BioPyhton* (electronic supplementary material, figure S1: *ND2*-based genetic distance matrices species).

Since reconstructing distant phylogenetic relations based on a single gene is unreliable [27,28], to create phylogenetic trees we used a published global bird database [23]. We used this database to create 100 bootstrapped trees for each set of species (congener: electronic supplementary material, figure S2; random sample: electronic supplementary material, figures S3 and S4). For our random and combined test sample (since for several species *ND2* was not available), we obtained the pairwise genetic distances from the bootstrapped trees using the *cophenetic* function of *phytools*. After obtaining the pairwise distance matrices for each of 100 trees, we averaged these 100 matrices to obtain a back-estimate of pairwise genetic distance.

### (c) Acoustic analysis

#### (i) Preprocessing

From each birdsong recording, we manually selected examples with minimal environmental noise and cut them into individual vocalizations using *Praat* [29]. We aimed to include at least two, maximally six, exemplar vocalizations for each species (table 1). Our final sample consisted of 315 vocalizations from 71 songbird species and 257 vocalizations from 66 non-songbird species. To remove potential noise, we performed band-pass filtering between 100 Hz and 10 kHz. We statistically standardized each acoustic sample (converted to floating-point values) using Python code to mean 0 and s.d. ± 1.0, before analysis, to account for potential amplitude differences between recordings.

#### (ii) Acoustic feature analysis

We extracted acoustic parameters from each vocalization using Python 3 and the *librosa* Python package [30], along with custom code. The acoustic feature analysis was based on short-time Fourier transforms (fft length: 512; hop length: 256; sampling rate: 22050 Hz). We selected nine acoustic features, sensitive to various spectral and temporal characteristics of the signal, extending the methods of a previous study demonstrating phylogenetic influences in anuran vocalizations [13]: root-mean-squared (RMS), spectral flux, spectral entropy, spectral flatness, spectral centroid (similar to dominant frequency) and spectral contrast in four frequency bands (contrast 1: 0–500 Hz; contrast 2: 500–1000 Hz; contrast 3: 1000–2000 Hz; contrast 4: 2000–4000 Hz). Spectral flatness indicates the tonality versus noisiness of a signal, on a gradient from 0 for white noise (equal energy at all frequencies) to 1.0 for a purely harmonic sound (all energy concentrated in partials). Spectral entropy is an information-theoretic measure, summarizing the overall spectral variability of the signal. By contrast, RMS, spectral contrast and spectral flux are more sensitive to local temporal variation in amplitude and spectrum across a signal [13,30]. Finally, treating the normalized amplitude spectrum as a distribution of energy over frequencies, the spectral centroid is the weighted mean of the distribution. Intuitively, this is the centre of mass of the distribution, where it would balance on your fingertip. We also calculated the traditional measure, dominant frequency, using custom code, but since this was highly correlated with spectral centroid, only the latter was included in our phylogenetic analyses.

These features were pre-selected based on prior published work, and the parameters of the acoustic analysis were not changed *post hoc* after phylogenetic signal calculation. After calculating the values of each of these features for each vocalization, we averaged across vocalizations for each species (see electronic supplementary material, figure S5 for acoustic data on each species and feature). To obtain acoustic distances, we calculated the mean absolute difference between the mean measures across the different recordings for each of the nine features described above. To obtain an overall measure of acoustic distance, we normalized the acoustic distance matrix for each of the acoustic features, then averaged the nine normalized distance matrices.

#### (iii) Acoustic feature analysis details

RMS amplitude was calculated for each time-frame of the spectrogram using *librosa*. We then calculated the standard deviation of this temporal signal and divided it by the mean (making it a unit free measurement: a coefficient of variation). Spectral flux was calculated for each time-frame using custom code (squared difference of the spectrum), then we took the log (s.d.) of the spectral flux, calculated for each time-frame. Spectral entropy was calculated for the entire vocalization using the Entropy package (github.com/raphaelvallat/entropy). Spectral flatness was calculated using *librosa* for each time-frame, then averaged across time and log transformed. Spectral centroid was calculated for each time-frame of the spectrogram with *librosa*, then the logarithm of the median was calculated across time-frames, owing to the large differences in spectral centroid between species. Similarly, contrasts 1–4 were also calculated for each time-frame of the spectrogram, than averaged across the vocalization and log transformed. Code performing the acoustic analysis is freely available at https://github.com/jozsarato/PhyloBirdSong.

#### (iv) Phylogenetic influences on acoustics

Phylogenetic signals were calculated using the *phytools* package in R. All other statistical analyses were performed in Python, with statistics calculated using the *scipy* and *scikit-learn* libraries. To initially assess whether there is a relationship between acoustic measures and genetic distances, we used three types of analyses separately for our sets of vocal learner and non-learner species. First, we used traditional statistical techniques (*t*-tests, correlations, regression) to test if closely related species have similar songs, and to assess whether acoustic measures can predict genetic distances at the level of individual species. In the correlation analysis, we calculated Pearson's *r* between genetic distance and each of the nine acoustic distance measures, then we averaged the obtained *r*-values for each species. Second, at the group level, we used Mantel tests to test for a relationship between genetic and acoustic distance matrices (code: https://github.com/jwcarr/MantelTest), and then performed 5000 bootstrap permutations to obtain Mantel test *p*-values. Finally, we used phylogenetic measures (Bloomberg's *K* and Pagel's λ) to test for a relationship between the acoustic and genetic distance matrices at the group level. The benefits and pitfalls of Bloomberg's *K* and Pagel's λ remain debated [31–33], and they may well contain complementary information. We therefore calculated both measures for each of 100 bootstrapped trees (obtained from the birdtree.org website using ‘phylogeny subsets’), for each group of species, and present both the mean values and the proportions of significant (*p* < 0.05) trees.

Finally, since similarities in spectral centroid potentially result from body size differences, we gathered weight information from a public electronic database [34] based on Dunning [35]. We performed multiple regression analyses, using the nine acoustic feature distances combined with log body mass differences, to predict genetic distances species-by-species. Next, the obtained *β*-values were averaged across species, to estimate the predictive strength of each acoustic feature after controlling for body mass.

## 3. Results

### (a) Related species have similar vocalizations, particularly in passerines

**Figure 2.**
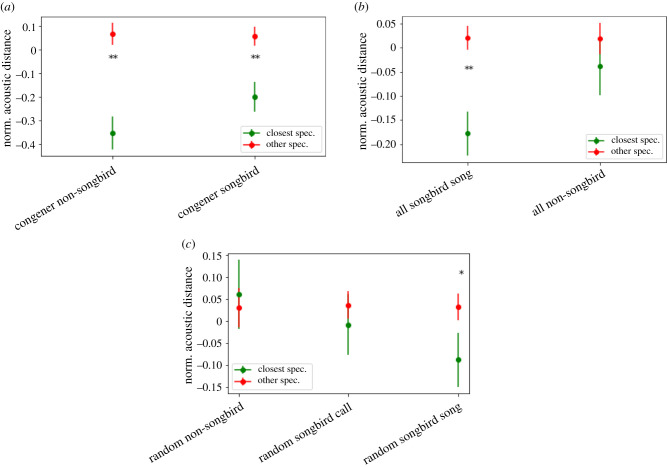
Acoustic distance between each species and its closest genetic relative (closest spec.) is smaller than the average across other species (other spec.) in our training set (*a*) for both passerines and non-passerines (*x*-axis). (*b*) In our random sample of species, this effect was only significant for passerine songs. (*c*) The effect was very strong in the combined sample of all passerine species in our dataset, but not significant for non-passerines (*p < .05, **p < .01, error bars: s.e.m.). (Online version in colour.)

Combined acoustic distance was smaller between each species and its closest genetic relative than the average of other species, for both passerine (*t*_27_ = 3.99, *p* < 0.001, figure 2*a*) and non-passerine (*t*_25_ = 4.88, *p* < 0.001, figure 2*a*) species in our congener set. This effect was absent in our stratified random sample of non-passerines (*t*_39_ = 0.426, *p* = 0.673, figure 2*b*) and stratified sample of passerine calls (*t*_36_ = 0.723, *p* = 0.474, figure 2*b*) but was present in random passerine songs (*t*_42_ = 2.194, *p* = 0.034, figure 2*b*). Finally, we found a strong effect for the combined sample of songs from 71 songbird species (*t*_70_ = 4.705, *p* < 0.001, figure 2*c*) but no effect for the combined sample of 66 non-songbird vocalizations (*t*_65_ = 0.984, *p* = 0.329, figure 2*c*). For a more detailed version of this analysis, see electronic supplementary material, figure S6, showing distance results for individual acoustic features. Thus, acoustic distance reflects genetic distance, at least for relatively close relatives, and particularly in passerine songs.

### (b) Species-wise analysis of acoustic–genetic distance correlations

The correlation between genetic and acoustic distances for each species of our congener set showed that, among songbirds, almost all species (24 out of 28, electronic supplementary material, figure S7*a*) show a positive correlation (mean *r* = 0.08, s.d. = 0.07). Similarly, most non-songbirds (20 out of 26, electronic supplementary material, figure S7*b*) also have an expected positive correlation (mean *r* = 0.08, s.d. = 0.16) between acoustic and genetic distances, with a higher variability across species.

### (c) Acoustic–genetic distance matrix similarity

Using the Mantel test on our training data, we found a significant correlation (*p* < 0.05) between the genetic distance matrix and that for 5 out of 9 acoustic features (table 2), as well as between genetic distance and the average acoustic distance matrix. However, in the random sample of songbird songs, there was only one significant feature (contrast 2: 500–1000 Hz), and two nearly significant features (spectral centroid, entropy), while no significant effects were found for songbird calls. In the random sample of non-songbirds, spectral centroid showed a strong effect, but no other measures were significant (table 2). In the combined set of all songbird species, we found a significant relationship for four acoustic features and the mean acoustic distance, while for the combined set of non-songbirds, only two acoustic features (spectral centroid, spectral flux) showed a significant relationship (table 2).

**Table 2.** Mantel test results for the measures described in the left column and genetic distances, for each dataset (columns). Bold: *p* < 0.05. RMS, root-mean-squared.

measure	dataset					
	congener set	random songbird song	random songbird call	random non-songbirds	combined songbirds	combined non-songbirds
mean acoustic distance	***r* = 0.211**	*r* = 0.062	*r* = −0.041	*r* = 0.079	***r* = 0.196**	*r* = 0.063
	***p* < 0.001**	*p* = 0.16	*p* = 0.743	*p* = 0.192	***p* = 0.001**	*p* = 0.117
RMS	*r* = 0.07	*r* = −0.106	*r* = −0.045	*r* = −0.071	*r* = 0.062	*r* = −0.03
	*p* = 0.142	*p* = 0.958	*p* = 0.792	*p* = 0.845	*p* = 0.124	*p* = 0.696
spectral flux	*r* = 0.031	*r* = −0.143	*r* = 0.062	*r* = 0.096	***r* = 0.167**	***r* = 0.093**
	*p* = 0.27	*p* = 0.986	*p* = 0.159	*p* = 0.137	***p* = 0.004**	***p* = 0.04**
entropy	*r* = −0.002	*r* *=* *0.117*	*r* = −0.045	*r* = −0.001	***r* = 0.092**	*r* = 0.041
	*p* = 0.508	*p* *=* *0.055*	*p* = 0.746	*p* = 0.468	***p* = 0.033**	*p* = 0.183
flatness	***r* = 0.116**	*r* = −0.031	*r* = −0.111	*r* = −0.08	*r* = 0.011	*r* = −0.033
	***p* = 0.018**	*p* = 0.652	*p* = 0.98	*p* = 0.828	*p* = 0.403	*p* = 0.742
spectral centroid	***r* = 0.206**	*r* *=* *0.104*	*r* = 0.088	***r* = 0.322**	***r* = 0.182**	***r* = 0.221**
	***p* = 0.001**	*p* *=* *0.054*	*p* = 0.114	***p* = 0.005**	***p* < 0.001**	***p* = 0.001**
contrast 1	*r* = 0.07	*r* = 0.074	*r* = 0.025	*r* = 0.009	*r* = 0.011	*r* = −0.035
	*p* = 0.09	*p* = 0.121	*p* = 0.298	*p* = 0.418	*p* = 0.382	*p* = 0.756
contrast 2	***r* = 0.16**	***r* = 0.112**	*r* = 0.029	*r* = 0.01	*r* = 0.076	*r* = 0.033
	***p* = 0.004**	***p* = 0.049**	*p* = 0.312	*p* = 0.418	*p* = 0.071	*p* = 0.204
contrast 3	***r* = 0.174**	*r* = 0.062	*r* = −0.029	*r* = −0.056	***r* = 0.084**	*r* = −0.053
	***p* < 0.001**	*p* = 0.131	*p* = 0.635	*p* = 0.734	***p* = 0.027**	*p* = 0.875
contrast 4	***r* = 0.112**	*r* = 0.038	*r* = −0.126	*r* = 0.105	*r* = 0.036	*r* = 0.03
	***p* = 0.024**	*p* = 0.26	*p* = 0.992	*p* = 0.121	*p* = 0.217	*p* = 0.242
body mass	***r* = 0.268**	*r* = 0.045	*r* = 0.029	***r* = 0.367**	***r* = 0.285**	***r* = 0.272**
	***p* = 0.0**	*p* = 0.201	*p* = 0.301	***p* = 0.001**	***p* < 0.001**	***p* < 0.001**

Bloomberg's *K* and Pagel's *λ* show strong phylogenetic influences in the training set for songbirds, but weak in non-songbirds.

**Figure 3.**
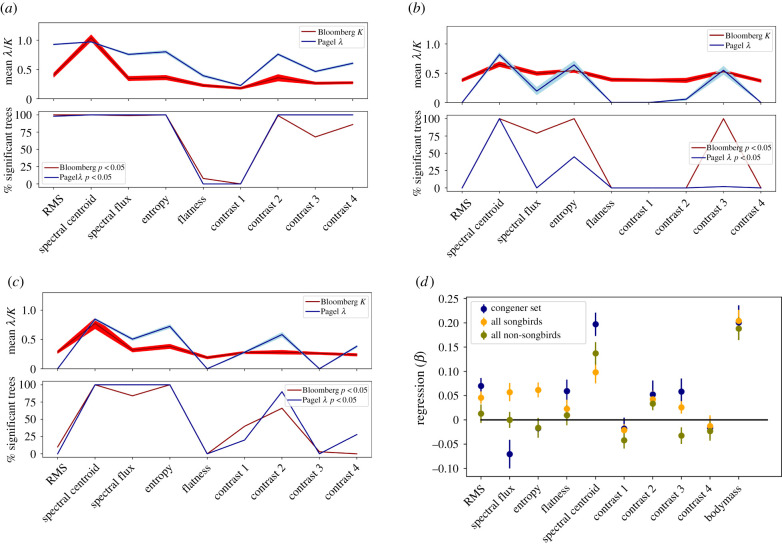
(*a*–*c*) Phylogenetic signal in the training set (*a*), in all passerine songs (*b*) and in all non-passerine vocalizations (*c*). Top panels show the average Pagel‘s *λ* and Bloomberg's *K* for each acoustic feature (*x*-axis) (shaded area: s.d.). Bottom panels show the per cent significant bootstrapped trees. (*d*) Combined prediction of genetic distance with control for body size: prediction of genetic distance from nine acoustic features and body mass (*x*-axis) in the training set and the combined set of passerine songs and non-passerines (see key, error bars: s.e.m.). (Online version in colour.)

In our training set, we found that seven out of nine acoustic features (figure 3*a*, all apart from flatness and contrast 1) show strong phylogenetic effects (almost all trees *p* < 0.05 with Bloomberg's *K*, and 100% of trees *p* < 0.05 with Pagel's *λ*). In the stratified sample of songbird songs (electronic supplementary material, figure S8*a*), four measures showed strong indications of phylogenetic signal (Bloomberg's *K* significant for 41–88% of trees, *p* < 0.05: spectral centroid, entropy, contrast 1 and contrast 3). For songbird calls (electronic supplementary material, figure S8*b*), two acoustic features showed indications of phylogenetic signal (contrast 1 and contrast 3, *p* < 0.05 for 100% of trees). In the random sample of non-songbirds (electronic supplementary material, figure S8*c*), spectral centroid was the only feature with significant phylogenetic signal (98% of trees *p* < 0.05). Calculating these measures for the combined sample of all songbirds (figure 3*b*) and all non-songbirds (figure 3*c*) species separately, we found remarkable similarities between the two groups. Spectral centroid, spectral flux and entropy showed phylogenetic signal for both groups (79–100% of trees *p* < 0.05), while no phylogenetic signal was detected in RMS and flatness (0% of trees *p* < 0.05). There was a difference in spectral contrast between the clades, as lower frequencies showed phylogenetic signal in non-songbirds (contrast 1–2: 0–1000 Hz, 40–66% of trees *p* < 0.05 with Bloomberg's *K*), but higher frequencies in songbirds (contrast 3: 1000–2000 Hz: 100% of trees *p* < 0.05 with Bloomberg's *K*).

### (d) Acoustic distance correlates with genetic distance controlling for body mass

Using body mass combined with acoustic features to predict genetic distance for each species, we found that despite controlling for body size, spectral centroid remains the strongest acoustic feature in predicting genetic distance. This was true in our congener set (*β*_Centroid_ = 0.197 ± 0.165), our combined set of songbirds species (*β*_Centroid_ = 0.098 ± 0.1), and also in non-songbirds (*β*_Centroid_ = 0.137 ± 0.137; figure 3*d*). Importantly, as expected, body mass strongly correlated with genetic distance (congeners *β*_Mass_ = 0.201 ± 0.257, all songbirds *β*_Mass_ = 0.204 ± 0.189, all non- songbirds *β*_Mass_ = 0.188 ± 0.192).

## 4. Discussion

Summarizing, in a large set of avian vocalizations including 71 songbird and 66 non-songbird species, using multiple measures, our results show that strong phylogenetic signal is present in vocally learned birdsong, as well as songbird calls and non-passerine display vocalizations presumed to be unlearned. Surprisingly, the magnitude of this phylogenetic effect for the learned song is comparable to—if not exceeding—phylogenetic influences on genetically determined vocalizations. We found the strongest phylogenetic effect with the simplest acoustic measure, spectral centroid, which summarizes the overall frequency distribution of the signal (as its weighted mean or centre of gravity). We also found strong phylogenetic effects with more complex measures (spectral contrast and flux), which are more sensitive to spectral variability, but not with others (spectral flatness and RMS). Whether the phylogenetic signal is truly absent from the latter measures remains an open question for future research, as larger samples, or acoustic analyses optimized for maximizing phylogenetic signal, might lead to different conclusions. In particular, using machine learning methods to fine-tune acoustic analyses to maximize phylogenetic signal for some training sample, and then deploying this on a much larger sample, would be a profitable way forward.

The phylogenetic signal estimates we obtained for our congener dataset, which included pairs of closely related species with small genetic distances, were larger than for our random stratified sample, and remained strong using the combined dataset including all species. This suggests that with the randomly selected species, with a much wider range of genetic and phenotypical variance, detection of phylogenetic influences is more challenging. Surprisingly, however, phylogenetic signal was strong and significant for passerine songs, but not calls, in the random dataset. This clearly goes against our expectation that songs, with a strong learned component, would exhibit weaker phylogenetic influences compared with (mostly unlearned) calls and undoubtedly deserves further investigation with a larger selection of species.

The persistence of phylogenetic signal even in learned vocalizations clearly suggests that vocal learning is compatible with genetic determination of and/or genetic constraints upon vocalization structure, as previously shown for a clade of vocal learning parrots [19]. Although our analyses cannot isolate any specific genetic factors, three possible explanations seem likely. First, broad morphological constraints (e.g. overall body size) might play a role in constraining some specific acoustic features, such as fundamental frequency (*f*_0_) or dominant frequency, owing to allometric constraints on the size of vocal organs. Second, genetic factors may constrain specific morphological features relevant to vocalization, such as details of syringeal morphology or of vocal tract structure [36–38]. Given the considerable well-known variability in syringeal anatomy across birds, we might expect acoustic measures tightly linked to anatomical variation to show strong phylogenetic signal, as reflected in the traditional use of syringeal anatomy in avian classification [39,40]. An investigation of this might be particularly revealing for clades like ducks, in which closely related species show wide variation in syringeal morphology the function of which is at present poorly understood [41,42].

Third, and most interestingly, there may be genetic constraints upon the neural control and/or learning mechanisms themselves, that is on the neural circuitry involved in selecting, imitating and producing vocalizations. This last possibility is particularly intriguing, and relevant for our dataset, given that the oscine syrinx, despite some limited variation [43], is a highly conservative structure, with its morphology shared by all of the nearly 4000 songbird species [40,44,45]. There is nonetheless huge variation in song acoustic structure among songbirds, and passerine mimics (mockingbirds, reed warblers, starlings or lyrebirds) can accurately copy songs of many species. Together, these facts suggest that an important source of genetic canalization in passerine song, reflected in the phylogenetic signal documented in the current study, concerns the wiring of the learning mechanism itself, rather than vocal morphology. This might be reflected in the details of the innate ‘sound template’ used by young birds to select their tutor(s) [46,47], and/or the ‘song grammar’ or syntax used to combine learned elements into higher order patterns [48–50]. This may also influence the acquisition and modification of song structure in adults, since it is now clear that open-ended song learners exhibit out-of-season reductions in song nuclei combined with adult neurogenesis, presumed to play an important role during learning of new songs [51,52], and are presumably under strong genetic control.

Clarifying the issue of the neural and morphological bases of phylogenetic constraints on song structure, and adjudicating among these possibilities, will constitute a massive research project, involving both more species and more complex analysis methods than those employed here. Nonetheless, we believe our current results clearly show that the methods and approach used here (along with other recent work, e.g. [19]) offer a promising route to further explore these fascinating questions. A richer understanding of the source(s) of phylogenetic constraints on learned and unlearned vocalizations is central to better understanding the evolution of vocal learning and of vocal communication more generally. Broad-scale genetic/acoustic analyses like those introduced here should play an important role in such future work.

## Data accessibility

Data and code supporting this publication can be found at https://github.com/jozsarato/PhyloBirdSong. The raw auditory data used for this publication is under copyright, please contact the authors if you need more information.
